# Induction of the pro-inflammatory NF-kB-sensitive miRNA-146a by human neurotrophic viruses

**DOI:** 10.3389/fmicb.2015.00043

**Published:** 2015-02-03

**Authors:** James M. Hill, Christian Clement, Yuhai Zhao, Walter J. Lukiw

**Affiliations:** ^1^Departments of Microbiology and Pharmacology, Louisiana State University Health Science CenterNew Orleans, LA, USA; ^2^LSU Neuroscience Center and Department of Ophthalmology, Louisiana State University Health Science CenterNew Orleans, LA, USA; ^3^Infectious Diseases, Experimental Therapeutics and Human Toxicology Lab, Department of Natural Sciences, Southern University at New OrleansNew Orleans, LA, USA; ^4^Department of Neurology, Louisiana State University Health Science CenterNew Orleans, LA, USA

**Keywords:** Alzheimer's disease (AD), aluminum sulfate, ebola virus, innate-immune response, messenger RNA (mRNA) and microRNA (miRNA), microRNA-146a, neuroinflammation, prion disease

A remarkably wide variety of human neurotrophic viruses—ranging from herpes simplex 1 (HSV-1; *Herpesviridae*; dsDNA genome) to Hantavirus (HTV; *Bunyaviridae;* (−)ssRNA genome) to human immunodeficiency virus (HIV; *Retroviridae*; (+)ssRNA genome) are associated with the rapid up-regulation of the NF-kB-sensitive pro-inflammatory microRNA-146a (miRNA-146a) in the host shortly after infection. This significant miRNA-146a up-regulation appears to be beneficial to the infecting virus as part of an immune-evasion strategy. Interestingly, miRNA-146a is also significantly up-regulated in several human central nervous system (CNS) disorders. These include Alzheimer's disease (AD) and prion disease where miRNA-146a participates in pro-inflammatory and innate-immune signaling. This opinion paper will comment on some recently clarified roles for the NF-kB-regulated, pro-inflammatory miRNA-146a in viral-induced cellular dysfunction, and how anti-miRNA-146a and/or related therapeutic strategies may be beneficial in the clinical management of a broad spectrum of viral-mediated CNS disease.

The 22 nucleotide, non-coding, single stranded RNA (ssRNA) miRNA-146a (5′-UGAGAACUGAAUUCCAUGGGUU-3′; 41% C+G; NR_029701) lies at the crossroads of multiple biological processes involved in the innate-immune response, viral-infection and inflammatory disease (Lukiw and Pogue, 2007; Cui et al., 2010; Lukiw, 2012; Saba et al., 2014). miRNA-146a, encoded at chromosome 5q33.3 (chr 5q33.3) in humans, is a rapidly induced, NF-kB-sensitive pro-inflammatory miRNA with a relatively short half-life of about ~2 h in the human CNS (Taganov et al., 2006; Lukiw et al., 2008; Sethi and Lukiw, 2009; Li et al., 2010; Kroesen et al., 2015). Initially described as being significantly up-regulated after microbial lipopolysaccharide (LPS) stimulation of monocytes and under transcriptional control by NF-κB, miRNA-146a was subsequently found to be: (i) up-regulated by pro-inflammatory cytokines (such as IL-1β and TNFα; Taganov et al., 2006; Lukiw et al., 2008; Cui et al., 2010); (ii) induced by metal sulfate-generated reactive oxygen species (ROS; Pogue et al., 2009); (iii) up-regulated by neurotoxic 42 amino acid amyloid beta (Aβ42) peptides in human primary brain cells (Li et al., 2010; Alexandrov et al., 2011); and (iv) implicated as a key regulator of innate-immune signaling in part through interleukin receptor-associated kinase (IRAK) activation (Cui et al., 2010; Saba et al., 2014). Subsequent sequencing across the chr 5q33.3 locus indicated the presence of 3 tandem, canonical NF-kB-binding sites in the 5′ regulatory-region of the miRNA-146a gene (Sethi and Lukiw, 2009; Cui et al., 2010). Combined with functionality and NF-kB-inhibition assays miRNA-146a was the first NF-κB-regulated, pro-inflammatory miRNA identified in the human CNS (Li et al., 2010, 2011). The most significant miRNA-146a abundances to date have been found in astroglial and microglial cells, the later representing the “resident scavenging-macrophages” of the CNS, and key participants in the brain's innate-immune surveillance and inflammatory response-systems (Li et al., 2010, 2011; Saba et al., 2012). While only basally expressed in the CNS, miRNA-146a can be induced 2- to 25-fold or higher in cultured human primary brain cells after the application of several different classes of physiological stressors including treatment with (i) neurotropic virus (Hill et al., 2009; Lukiw et al., 2009; Li et al., 2010); (ii) neurotoxic metal sulfates (such as aluminum sulfate at low nanomolar concentrations; Pogue et al., 2009); (iii) microbial endotoxins including LPS (Taganov et al., 2006); and (iv) pro-inflammatory cytokines and Aβ peptides, either alone or in combination (Taganov et al., 2006; Lukiw et al., 2008, 2010; Li et al., 2010). While the mechanism for miRNA-146a-mediated immune-evasion is still not fully understood, in humans this process appears to require the activation of NF-kB; other transcription factors such as AP1 may be used in miRNA-146a activation in mice (Tung et al., 2010; Ho et al., 2014; Wang et al., 2014).

A surprisingly large number of different types of potentially incapacitating or lethal viruses, possessing either DNA or RNA genomes, have been shown to significantly induce miRNA-146a in the human CNS, immune, lymphatic, hepatic or circulatory systems, and these include (alphabetically-ordered): (i) Chikungunya virus (CHIKV; *Togaviridae;* (+)ssRNA genome; Selvamani et al., 2014); (ii) enterovirus 71 (EV71; *Picornaviridae*; (+)ssRNA genome; Ho et al., 2014); (iii) Epstein-Barr virus (EBV; *Herpesviridae*; dsDNA genome; Jonigk et al., 2013); (iv) Hantavirus (HTV; *Bunyaviridae;* (−)ssRNA genome; Shin et al., 2013); (v) hepatitis C virus (HCV; *Flaviviridae*; (+)ssRNA genome; Joshi et al., 2013); (vi) herpes simplex virus-1 (HSV-1; *Herpesviridae*; dsDNA genome; Higaki et al., 2003; Hill et al., 2009; Lukiw et al., 2010); (vii) Henipavirus (Hendra) virus (HeV; *Paramyxoviridae*; (−)ssRNA genome; Stewart et al., 2013); (viii) human influenza A viruses (H1N1/H3N2; *Orthomyxoviridae*; (+)ssRNA genome; Chen et al., 2012; Terrier et al., 2013); (ix) hepatitis B virus (HBV; *Hepadnaviridae*; dsDNA genome; Liu et al., 2009); (x) human immunodeficiency virus (HIV; *Retroviridae*; (+)ssRNA genome; Duskova et al., 2013; (xi) human T-cell leukemia (lymphotropic) virus type 1 (HTLV-1; *Retroviridae*; (+)ssRNA genome; Pichler et al., 2008); and (xii) Japanese encephalitis virus (JEV; *Flaviviridae;* (+)ssRNA genome; Pareek et al., 2014). Note that (i) this viral-miRNA-146a-induction/association are all relatively recent discoveries with more than three quarters identified within the last 22 months; (ii) all of the most recent viral-host miRNA-nucleoplasmic signaling studies indicate the up-regulation of miRNA-146a; and (iii) viral infection involving *each virus mentioned above* is associated with progressive neuropathological change. Interestingly (i) miRNA-146a up-regulation has been associated with common age-related, human inflammatory degenerations such as sporadic Alzheimer's disease (AD), and the rare sporadic prion diseases Creutzfeldt-Jakob disease (sCJD) and Gerstmann-Straussler-Scheinker (GSS) syndrome; and (ii) the etiopathogenesis of AD has recently been associated with multiple viral infections, and most recently with latent HCV, HIV-1 or HSV-1 reactivation (Hill et al., 2009, 2014; Lukiw et al., 2011; Ball et al., 2013; Alexandrov et al., 2014).

Under suitable physiological conditions, often within minutes after viral infection, signaling via the pre-existing, heterodimeric transcription factor NF-kB is accomplished by complex, highly interdependent, viral-mediated regulatory mechanisms. These involve protein-protein interactions, phosphorylation, ADP-ribosylation, nucleocytoplasmic-trans-location, ubiquitination and proteolytic-degradation (Vallabhapurapu and Karin, 2009; Cui et al., 2010; Lee and Covert, 2010; Yarbrough et al., 2014; Di Girolamo, 2015). The most ubiquitous NF-κB members in non-stimulated cell cytoplasm are the p50 and p65 (RelA) subunits forming the heterotypic p50/p65 NF-kB dimer complexed with members of the IκB-inhibitor family (which prevents nuclear translocation; Zanella et al., 2013; Di Girolamo, 2015). Typically, after viral-mediated phosphorylation of IκBα at specific serine residues, IκBα dissociates from the p50/p65 dimer, is ubiquitinated and degraded by the proteasome, allowing the majority of NF-κB complexes to translocate through the nuclear pore complex (NPC; typically 10,000 nuclear pores/neuron; Threadgold, 1976). Here NF-kB subsequently recognizes genomic NF-kB binding sites in target gene regulatory regions, to transiently activate RNA Pol II-mediated transcription (Vallabhapurapu and Karin, 2009; Cui et al., 2010; Zanella et al., 2013; Di Girolamo, 2015). The miRNA-146a gene for example may be up-regulated 10-fold or more within minutes of viral infection; importantly NF-κB activation is usually terminated via IκB protein re-synthesis and NF-kB-re-inhibition (Schmid and Birbach, 2008; Hill et al., 2009, 2014; Cui et al., 2010; Lukiw et al., 2010). Gel shift assays and live-cell fluorescence microscopy indicate that NF-κB activation may exhibit oscillatory patterns, with levels of nuclear NF-κB alternately increasing-and-decreasing; this suggests the intriguing possibility that NF-kB-based signaling might exploit the timing of protein-modification and nucleocytoplasmic shuttling to regulate gene expression (Spiller et al., 2010; Kodaman et al., 2014). Oscillatory variation in miRNA-146a abundance is not well understood, indeed viral-mediated phosphorylation of IkB and NF-kB activation and nucleocytoplasmic trafficking is complicated as different viruses may recruit different viral or host proteins to target different signaling components of the NF-kB pathway using multiple strategies. For example polyubiquitination of the (+)ssRNA HTVL-1 virus encoded Tax protein activates IkB kinase resulting in NF-kB activation and nucleocytoplasmic translocation, while the dsDNA EBV-encoded latent membrane protein 1 (LMP1) not only activates IkB kinase to induce nucleocytoplasmic trafficking of NF-kB but also appears to be involved in additional mechanisms including LMP1-mediated interaction with nuclear proteins (Currer et al., 2012; Ersing et al., 2013). Interestingly, many neurotrophic viruses inhibit nucleocytoplasmic trafficking of host mRNAs to promote cytoplasmic viral replication and disrupt expression of anti-viral factors by the host (Yarbrough et al., 2014). What is remarkable is that despite a tremendous variation in their biophysical and genomic structure, nucleic acid type, size and life-cycle, in humans all miRNA-146a-inducing neurotrophic viruses appear to share the common capabilities: (i) to target NF-kB-mediated gene expression; (ii) to induce complex nuclear and/or nucleocytoplasmic signaling that processes miRNA-146a precursors to export mature miRNA-146a back into the cytoplasm; and (iii) to drive a miRNA-146a-mediated arachidonic acid signaling cascade with subsequent pro-inflammatory and pathogenic consequences (Hill et al., 2009; Lukiw et al., 2010; Alexandrov et al., 2014; Yarbrough et al., 2014).

Out of about 24,000 miRNAs so far identified in all species, only about 300 are encoded by viruses (miRBase v.20; Liu, 2014). There is evidence that viral-encoded miRNAs regulate the expression of their own genes or the host's genes, or both (Liu, 2014; Yao and Nair, 2014). dsDNA viruses encode most of the viral-encoded miRNAs, with members of the family *Herpesviridae* accounting for the vast majority, indicating the significance of viral miRNA-mediated gene regulation in the biology of HSV infection (see above; Yao and Nair, 2014). In general DNA viruses that contain miRNA encoded in their viral DNA require access to the RNA polymerase II and miRNA processing machinery located within the nucleus in order to express that miRNA. In contrast, RNA viruses can replicate in the cytoplasm and, therefore, rarely encode miRNA (Liu, 2014; Swaminathan et al., 2014; Yao and Nair, 2014). There are, however, notable exceptions - for example infection with Ebola virus [EBOV; *Filoviridae*; (−)ssRNA genome] that causes a highly lethal hemorrhagic fever syndrome in humans rapidly induces 3 EBOV genome-derived miRNAs that subsequently target host mRNA (Liang et al., 2014). Indeed, perhaps as part of complex survival and immune-evasion strategies, neurotrophic viruses may modulate host miRNA precursor processing to favor viral miRNA production, thus contributing to viral-disease pathogenesis via multiple and highly interactive mechanisms (Conrad and Niepmann, 2014; Liu, 2014; Yao and Nair, 2014; Yarbrough et al., 2014).

In summary, viruses have evolved multiple and complex strategies to subvert and evade the host immune-response to ensure their own replication and survival (Hill et al., 2014; Kodaman et al., 2014; Yarbrough et al., 2014). While there is still debate as to whether up-regulated miRNA-146a is beneficial to the infecting virus or a protective host innate-immune response, at least 7 recent observations suggest that a virally-induced NF-kB-mediated up-regulation of miRNA-146a is significantly pathogenic and disruptive to homeostatic CNS function: (i) the antiviral acycloguanosine acyclovir prevents an HSV-1-induced miRNA-146a-activated pro-inflammatory cell-death program in human CNS cells via reduction in miRNA-146a abundance (Lukiw et al., 2010); (ii) up-regulated miRNA-146a has been shown to significantly down-regulate expression of complement factor-H to induce a progressive and lethal pro-inflammatory degeneration in stressed human primary brain cells (Cui et al., 2010; Alexandrov et al., 2014); (iii) both viral and cytokine (IL-1β, TNFα) induced up-regulation of miRNA-146a triggers a chronic human retinal-degeneration (Kutty et al., 2013; Alexandrov et al., 2014; Hill et al., 2014); (iv) a progressive up-regulation of miRNA-146a accompanies pro-inflammatory neuropathology in lethal human CNS disorders including sporadic AD and the human-prion diseases GSS and sCJD (Lukiw et al., 2011; Saba et al., 2012); (v) a progressive up-regulation of miRNA-146a accompanies AD-type neuropathology in several transgenic animal models of AD (including Tg2576 and 5xFAD; Alexandrov et al., 2011, 2014; Li et al., 2011); (vi) quenching of miRNA-146a using anti-miRNA-146a strategies restores homeostatic immune signaling in CHIKV-infected human fibroblasts (Selvamani et al., 2014); and (vii) inhibition of EV71-induced miRNA-146a-upregulation employing anti-miRNA-146a strategies has been observed to inhibit viral propagation and improve survival rates in mouse models (Ho et al., 2014). It is our opinion: (i) that NF-kB inhibition may not be an effective therapeutic strategy for neurotrophic viral infections because NF-kB is a ubiquitous transcription factor with large potential for off-target effects; and (ii) that virally-induced miRNA-146a excess could be effectively neutralized using perfectly complementary locked nucleic acid-stabilized anti-miRNA-146a oligonucleotides, and thereby act as an anti-viral agent for a wide variety of DNA- and RNA-virus-induced disease (Lukiw, 2013; Maguire et al., 2014). Indeed, a major advancement in antiviral therapy might involve a broad-spectrum, anti-miRNA-146a strategy which, perhaps in combination with antivirals such as acyclovir and/or the recently described gene editing methods using CRISPR/Cas9 (clustered regularly interspaced short palindromic repeats/caspase 9)-mediated or other gene therapy technologies (Doudna and Charpentier, 2014; Maguire et al., 2014; Hochstrasser and Doudna, 2015). We envision these to have considerable therapeutic potential in the future clinical management of viral infections where miRNA-146a up-regulation appears to play a pathogenic role.

## Conflict of interest statement

The authors declare that the research was conducted in the absence of any commercial or financial relationships that could be construed as a potential conflict of interest.
